# Microstructural, Textural, Sensory Properties and Quality of Wheat–Yam Composite Flour Noodles

**DOI:** 10.3390/foods8100519

**Published:** 2019-10-21

**Authors:** Kai-Nong Sun, Ai-Mei Liao, Fan Zhang, Kiran Thakur, Jian-Guo Zhang, Ji-Hong Huang, Zhao-Jun Wei

**Affiliations:** 1 School of Food and Biological Engineering, Hefei University of Technology, Hefei 230009, Chinazffs2012@mail.ahnu.edu.cn (F.Z.); kumarikiran@hfut.edu.cn (K.T.); zhangjianguo@hfut.edu.cn (J.-G.Z.); 2 College of Biological Engineering, Henan University of Technology, Zhengzhou, 450001, China; aimeiliao@haut.edu.cn (A.-M.L.); huangjihong@haut.edu.cn (J.-H.H.); 3 Henan Cooperation Science and Technology Institute, Zhengzhou, 470001, China; 4 Anhui Province Key Laboratory of Functional Compound Seasoning, Anhui Qiangwang seasoning Food Co., Ltd., Jieshou 236500, China

**Keywords:** wheat flour noodles, Chinese yam, texture and rheological properties, microstructure, protein–gluten network

## Abstract

Herein, feasibility of supplementing wheat flour with Chinese yam powder (CYP) for noodle preparation was assessed. After supplementation with CYP, the alterations in chemical, texture, cooking, rheological, and microstructure attributes of noodles were observed. Due to higher protein and lower gluten, 20% of CYP promoted the stable network of gluten and starch particles. However, the excessive addition reduced the flexibility and the chewiness. The adverse changes were observed at 40% substitution level in texture profile analysis (TPA) and rheological parameters due to disrupted gluten–protein network which accelerated the exposure of starch particles. The CYP incorporation up to 20% showed better mouthfeel but further addition lowered the total sensory scores. Scanning Electronic Microscopy (SEM) analysis confirmed the modifications in noodles microstructure as CYP addition affected starch granule structure. In general, 30% substitution significantly improved the textural and rheological properties of noodles, indicating the potential of Chinese yam powder for industrial application.

## 1. Introduction

Noodles are rich in nutrition, easy to cook, and delicious traditional foods in Asian countries besides their increasing demand in many Western countries [1]. Traditionally, wheat flour has been mainly used for noodles preparation. However, several varieties of noodles have been reported which were prepared by using rice, buckwheat or starches derived from potato, sweet potato, and pulses [2,3,4]. Increasing economic development and awareness for healthy lifestyle among the consumers, emphasize noodles which are enriched with natural protein and other functional components derived from plants sources [5].

In the past years, researchers have been engaged to enrich noodles by fortifying them with various ingredients. The attempts have been made to use wheat noodles as a resource and then supplement them by adding broken rice, oat flour, barley flour, sweet potato flour, and buckwheat flour [1,2,6,7]. The recent trend to include vegetable materials to the wheat flour for the preparation of noodles has resulted in different flavors, colors, and additional nutrients with value added benefits [8].

Yam is recognized as a traditional staple food in many tropical regions in the world particularly in South Asia [9]. Chinese yam, *Dioscorea Polystachya* (Turczaninow)*,* is one of the important edible starch plants widely cultivated in China [10] and it is recognized as pharmaceutical food [11] with general composition (dry-weight basis) of 75–84% starch, 6–8% crude protein, and 1.2–1.8% crude fiber [12]. Yam tuber is an important nutritional food which contains many functional components such as dioscin, diosgenin, allantoin, choline, and proteins [12]. Among the popular Yam varieties in China, Chinese yam is a special variety available in Henan province with more functional components such as protein and polysaccharides [8]. 

Previous studies were conducted to evaluate the functional properties of noodles and the effects of various compounds to improve their final quality [5]. Noodle quality is assessed as a combination of appearance, texture, and cooking properties [13]. However, the information about the incorporation of Chinese yam flour for the preparation of wheat flour noodles is limited. The objective of this study was to investigate the physicochemical properties of noodles and properties of dough when flour was partly substituted by white yam flour. Our results can offer essential hints for the increasing of nutritional value of noodles and the development of noodles derived from the mixture of wheat and yam flours.

## 2. Materials and Methods

### 2.1. Raw Materials

Wheat flour (Golden dragon fish brand, Yihai Kerry, Shanghai, China) and table salt were purchased from Carrefour supermarket in Hefei city, China. The dried Chinese yam thin piece was procured from Tanan Local Wholesale Shop (Jiaozuo, China). At the laboratory, Chinese yam powder (CYP) was ground using Xichu Food Grinder (Jinhua, China), and passed through 100 mesh sieve.

### 2.2. Physicochemical Characterization of Wheat Flour and CYP

#### 2.2.1. Chemical Composition

The proximate composition (contents of ash, moisture, protein, fat, and gluten) of purchased wheat flour and CYP were tested by using the standard methods of Association of official analytical chemists (AOAC) [14]. Ash content was determined according to International association for cereal science and technology (ICC) [15] and its starch content was determined according to Food and agricuilure organization of the United Nations (FAO) [16]. All the measurements were performed in triplicates and the results were expressed as means ± standard deviations.

#### 2.2.2. Starch Gelatinization Properties

The starch gelatinization properties of wheat flour supplemented with different concentrations (0%, 10%, 20%, 30%, and 40% by dry weight basis) of CYP were determined by Differential Scanning Calorimetry (DSC) [17]. Briefly, 3.0–5.0 mg of yam-supplemented wheat flour was placed in aluminum crucible and distilled water was added (1:2 *w*/*w*). The aluminum crucible was stored under refrigerated conditions (4 °C) for 12 h. The empty crucible was taken as control. Initially, the starting temperature of the sample was 30 °C. After holding at 30 °C for 1 min, sample was heated to 90 °C at a rate of 10 °C/min [18]. The following thermodynamic parameters were recorded:

Tp: gelatinization temperature peak; To: onset gelatinization temperature; Tc: conclusion gelatinization temperature; R: gelatinization temperature range. R was calculated following Formula (1).

R = Tc − To(1)

#### 2.2.3. Farinograph Properties

According to a previous study with few modifications [11], the farinograph properties were determined using Farinograph-E (Brabender, Duisburg, Germany). For this, 300 g wheat flour was supplemented with different levels (0%, 10%, 20%, 30%, and 40% by dry weight basis) of CYP and placed in the mixer. A certain amount of water was added into the mixer and each dough was stirred in the mixer until the consistency of 500 ± 20 flux unit was achieved. In order to evaluate the quality of dough, the following farinograph factors were recorded from the farinograph curve: Water absorption, Dough development time, Dough stability time and Degree of softening. All the measurements were performed in triplicates and the results were expressed as means ± standard deviations.

#### 2.2.4. Rheological Properties

Rheological evaluation, especially in the linear viscoelastic region, has been used for unraveling the structure and properties of dough in order to study the functions of dough ingredients [19]. Elasticity and viscosity are among the important parameters of rheological attributes. The resulting doughs from supplemented wheat flour with different levels of CYP were tested with a steady-shear rate ranging from 0.01 to 1000^−1^ at 20 °C and as a function of frequency (0.1 to 10 Hz) using a Discovery Hybrid Rheometer-3 (TA instruments, New Castle, DE, USA) equipped with a cone-and-plate geometry (diameter 40 mm, cone angle 2°). All parameters were tested as per the above-mentioned experimental conditions.

### 2.3. Noodles Preparation

Based on an earlier study [20], noodles were prepared using yam (0%, 10%, 20%, 30%, and 40% *w*/*w*)-supplemented wheat flours mixed with 50% distilled water, 2% NaCl, and 0.1% NaHCO_3_. To prepare the dough, 100 g of supplemented powder (different yam levels) was mixed with required amount of water in an automatic noodle machine (Joyoung, Hangzhou, China). The dough was sheeted to about 2 mm thickness and the resulting fresh noodles were cut into 15 cm.

### 2.4. Cooking Properties

For this, noodles (15 cm) strips were cooked for 2 min. According to the method of Lu et al. (2009) with few modifications [21], cooking yield and cooking loss were determined. Briefly, ten noodles were weighed (W_1_) and put into a beaker with 400 mL of boiled water. After cooking for 2 min, the noodles were allowed to cool down by placing them in cold water for 30 s followed by drying for 3 min and weighed (W_2_). The cooking yield of the noodles was calculated using the following Formula (2)

Y (cooking yield) = W_2_/W_1_ (2)

W_2_ = Weight of cooked noodle after drying; W_1_ = Weight of raw noodle.

For the cooking loss, the final weight (W_3_) is recorded as the weight of beaker after drying. The cooking loss of the noodles was calculated using the following Formula (3)

Y (cooking loss) = (W_3_ − W_4_)/W_1_ (3)

W_4_ = Weight of beaker; W_3_ = the weight of beaker after drying; W_1_ = Weight of raw noodle.

### 2.5. Tension Properties and Texture Profile Analysis (TPA)

According to the method of Sangpring (2015) [22], three noodles were cut into 6 cm length and then placed on the metal plate. Samples were tested three times using A/KIE probe at Tension pattern. The pre-test speed was set up at 2 mm/s, the test speed was 1 mm/s, the post-test speed was 2 mm/s, the stretching distance was 12 mm, the induction force was 5 g, and the trigger point was 10 g. The tensile curve was obtained at the end of experiment. Two tension parameters were obtained from the curve: maximum extensibility distance and extensibility resistance force. Another three samples were tested three times to 90% of original sample thickness using R/36R probe at TPA pattern and programming was set the same as mentioned in Tension pattern. Four TPA parameters were obtained from the force–time curve: hardness, adhesiveness, springiness, and cohesiveness.

### 2.6. Sensory Properties

For sensory evaluation, five types of noodles samples (100 g) were cut into 15 cm length and cooked up to their optimal time (the time when white core in the cross section disappeared) in 100 °C water. The samples were dried for not more than 10 min in tightly covered plastic food containers before testing. Sample noodles were evaluated by 20 students and staff of the Food Science and Engineering College, Hefei University of Technology. All the samples were coded in a randomized order and evaluated by a 9-point hedonic scale (1: dislike extremely, 5: neither like nor dislike, 9: like extremely). The different sensory attributes included color, appearance, taste, flavor, hardness, toughness, and overall acceptability. 

### 2.7. Microstructure of Noodles

The raw and cooked samples were cut into 3 cm and frozen at −80 °C. After freeze-drying, the dried samples were cut into cross-section and placed on a specimen holder for coating with gold for 90 s. The microstructure of samples was observed by Scanning Electron Microscope (JEOL USA, Boston, MA, USA), at 22 KV with 250× and 1000× magnification.

### 2.8. Statistical Analysis

All the measurements were performed in triplicate and the obtained results were expressed as the mean values ± standard deviation (SD). The results were statistically analyzed using SPSS (SPSS Inc.; Chicago, IL, USA). For the data analysis, one-way analysis of variance (ANOVA) was performed using the Origin Lab (Origin Pro 8.0, OriginLab, Northampton, UK) software.

## 3. Results and Discussion

### 3.1. Characterization of Wheat Flour Supplemented with CYP

The chemical compositions of original wheat flour, CYP, and the mixed flour with different amounts of CYP was presented in Table 1. It can be seen clearly that there was no significant difference in ash content among different samples which indicated that similar inorganic impurities in wheat flour and CYP. The content of protein and starch increased with the rising proportion of CYP, whereas the contents of moisture, fat, and gluten decreased. Initially, protein content in the mixed flour ranged from 8.55% (wheat) to 12.49% (yam). Likewise, starch content ranged from 68.3% to 72.2%. These results were consistent with the previous study where protein content ranged from 11.94% to 13.00% with the lowest yam supplementation (95% wheat flour: 5% three-leaf-yam flour) and the highest yam supplementation (85% wheat flour: 15% three-leaf-yam flour), respectively. Inclusion of three-leaf-yam flour increased the protein content in wheat composite [23]. As previously known, yam is a good source of protein, polysaccharides which can contribute to the support of the protein–gluten network [23]. However, at the same time protein can compete with starch for water. Therefore, absence of gluten in CYP may weaken the elasticity of dough. 

### 3.2. Starch Gelatinization Properties

At first, lower gelatinization peak temperature in wheat flour was observed (*p* < 0.05) as compared to CYP. From Table 2 and Figure S1, it can be seen that with the addition of CYP, onset gelatinization temperature gradually increased from 59.42 °C to 72.45 °C, the conclusion gelatinization temperature gradually increased from 72.24 °C to 82.68 °C, and gelatinization temperature range showed a downward trend which was consistent with a previous study [17]. Moreover, earlier studies proved that gelatinization temperature is proportional to change in starch properties (structure of starch and starch crystallinity) [24]. As reported previously, the gelatinization peak temperature increased in the presence of protein and starch [25]. Similarly, our results also explained that CYP could influence the combination and crystallinity of starch. At the same time, more protein content could reduce the water activity of flour and ultimately caused the gelatinization hardening of the flour.

### 3.3. Farinograph Properties

From Table 2, it was obvious that the addition of CYP displayed effects on the farinograph properties of dough. Dough development time is relative to water absorption rate. Our results showed that the dough development time increased with the increased water absorption rate. The water absorption of the wheat flours supplemented with CYP increased significantly when CYP content increased from 0% to 30%. Dough development time of the supplemented wheat flours increased significantly when CYP content increased from 10% to 30%. Similar changes were observed by Zaidul et al. (2010) [26] on blending sago with wheat flour, and black rice with wheat flour. This can be explained by different characterization of wheat flour and CYP. The polar group in protein can combine with water through hydrogen-bond interaction and physical entrapment which can enhance the water absorption rate. At the same time, protein and some polysaccharides can help to fill protein–gluten network which can influence the dough development time, dough stability time, and degree of softening. 

Dough stability time is an important index for wheat flour to represent the gluten properties. The less time required for dough formation can be related to the decreased gluten content. Our results showed that the addition of CYP resulted in decreased gluten in blends and weakened the expansion of gluten network. If gluten protein is removed from the wheat flour, the flour is likely to lose its stability. Higher concentrations of CYP can weaken the amount of gluten which was the reason for disintegration of the dough. Almost all the farinograph properties were changed evidently at 40% CYP supplementation. Higher concentration of CYP can cause the dough to fail to support the starch granule to bond in the protein network and make the dough unstable. The above finding was supported by the previous study where the addition of fenugreek resulted in a more stable dough due to formation and stabilization of the gluten network structure [27].

### 3.4. Rheological Properties

**Figure 1:**
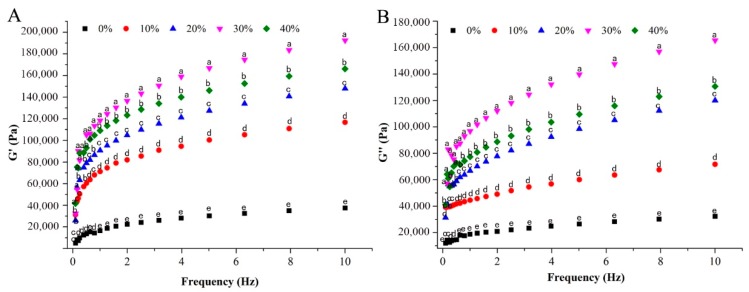
Effects of wheat flour supplemented with different levels of Chinese yam powder (CYP) on rheological properties. Storage moduli (G’) (**A**) and Loss moduli (G’’) (**B**). The values of different letters represent the significantly different (*p* < 0.05) of wheat flour supplemented with different levels of CYP at the same frequencies.

The rheological properties of dough were characterized in terms of viscoelasticity and viscosity. Figure 1A displays the storage moduli (G’) and Figure 1B shows loss moduli (G’’) of samples as a function of frequency which described the elasticity and viscosity of the dough. Both of G’ and G’’ of samples increased with the increasing addition of CYP up to 40%. The high protein and polysaccharide in CYP could promote association of starch molecules resulting in a bridging effect [24]. However, further addition can disturb protein–gluten network unable to support the starch granule which resulted into reduced elasticity and viscosity of the tested samples.

### 3.5. Cooking Quality

**Figure 2:**
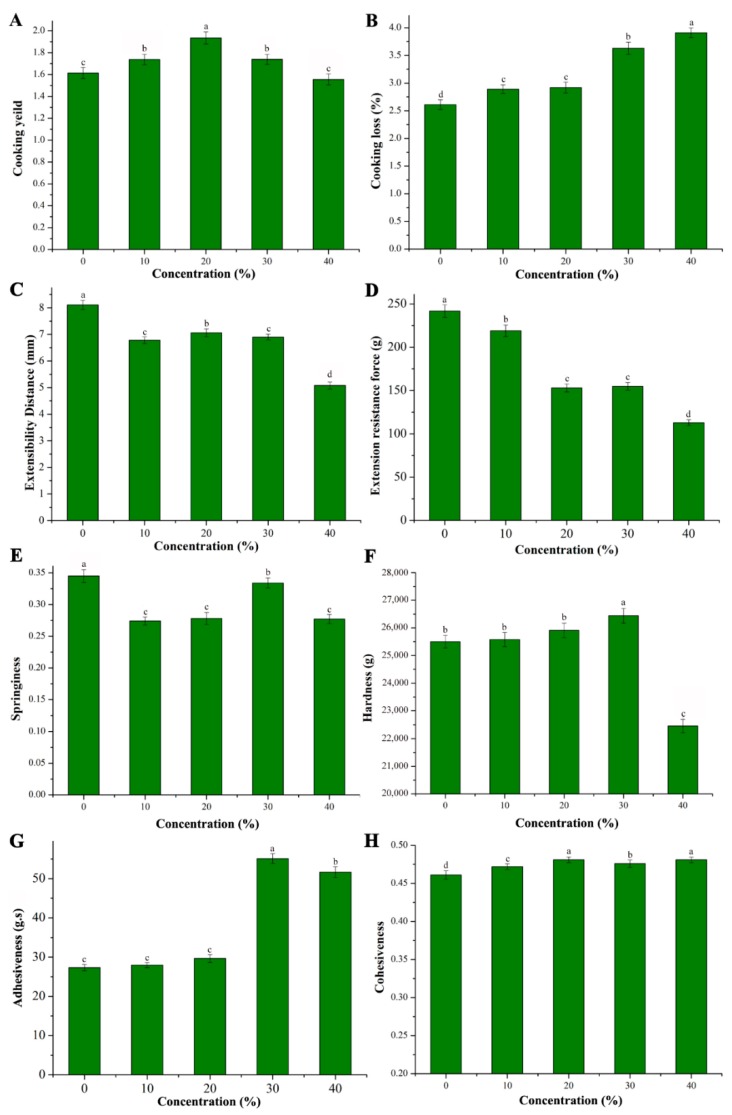
Effects of wheat flour supplemented with different levels of Chinese yam powder on cooking properties, tension properties, and texture properties. Cooking yield (**A**), Cooking loss (**B**), Extensibility distance (**C**), Extensibility resistance force (**D**), Springiness (**E**), Hardness (**F**), Adhesiveness (**G**), Cohesiveness (**H**). The values followed by different letters were significantly different (*p* < 0.05).

Cooking loss and cooking yield are two important parameters of cooking quality. The cooking yield was defined as the percentage of noodle weight after cooking to the weight of raw noodles, hence it represented the ability of the noodles to absorb water from the cooking medium. The cooking loss represented the particles that diffused out from the noodles into the cooking medium during cooking [2]. High cooking yield and low cooking loss are essential for noodles. The cooking yield increased with the increased addition of CYP prior to 30% (Figure 2A). Relatively higher cooking yield may indicate more starch and protein of CYP. Due to the higher amount and amylose content in CYP, starch is likely to expand when mixed with water after the enlargement in the protein–gluten network. However, after the addition of 30% CYP, the cooking yield was decreased which might be due to the loss of starch grain [28].

In Figure 2B, cooking loss of CYP showed that the noodles prepared with wheat flour supplemented with CYP had significantly higher cooking loss values as compared to non-supplemented wheat flour noodles. This might be due to the fact that polymer interaction in protein–gluten network can encapsulate the starch granules. Nevertheless, competing for water, protein can speed up the loss of starch [4]. Previous studies also demonstrated that cooking loss could be due to the disruption of the protein starch matrix through diluted gluten fraction in case of oat flour [29]. 

### 3.6. Tension and Texture Properties

The extensibility distance and extensibility resistance are two measures of tensile curve. Extensibility distance relates to the extensibility of the noodles and extensibility resistance mainly refers to palatability and chewiness. The extensibility distance and extensibility resistance have the same downward trend altogether as shown in Figure 2C,D. It indicated that the addition of CYP can decrease the elasticity in particular. However, a certain amount of CYP can increase the elasticity of noodles. At the addition of 30% CYP, better elasticity was achieved and on the other hand, 40% of CYP supplementation clearly destroyed the tension properties of noodles. This result was confirmed by comparing with other properties in our study. Better elasticity could be due to the co-action of protein and polysaccharides and starch gelatinization. At the same time, it can also be supported by texture profile analysis [30]. 

Hardness, springiness, adhesiveness, and cohesiveness are the four important parameters used during texture profile analysis (TPA). The addition of CYP decreased the springiness of wheat flour noodles which might be due to decreased gluten (Figure 2E). No gluten in CYP weakens the co-action of starch and protein network. On the other hand, increasing concentration (up to 30%) of CYP could significantly enhance the springiness from 0.274 to 0.334. In a previous study, addition of 7% fenugreek can enhance the springiness of wheat flour noodles [20], because the high level of elasticity of wheat dough is generally conferred by gluten [31]. The increased protein can promote the co-action with polysaccharides to increase the springiness.

The hardness initially increased from 25,504.35 g to 26,440.21 g after the addition of CYP (up to 30%) (Figure 2F) which may be due to the fact that increased protein can lead to the decreased water absorption of gluten network. These findings were consistent with the previously reported study of Sulieman et al. [32]. Polysaccharides can interact with protein or starch, making functional replacement of gluten network to impart firmness to noodles [31]. After the addition of 40% CYP, the hardness was decreased.

It can be seen from the Figure 2G, that adhesiveness significantly increased at the addition of 30% CYP. The rich protein in yam is responsible for the quick hydration and absorption of water which results into high viscosity [33]. Little significant differences were observed for cohesiveness values of noodles with addition of CYP (Figure 2H). As cohesiveness is an indication of how the components hold together, CYP appeared with no obvious effect to the cohesiveness values of noodles.

### 3.7. Sensory Properties

**Figure 3:**
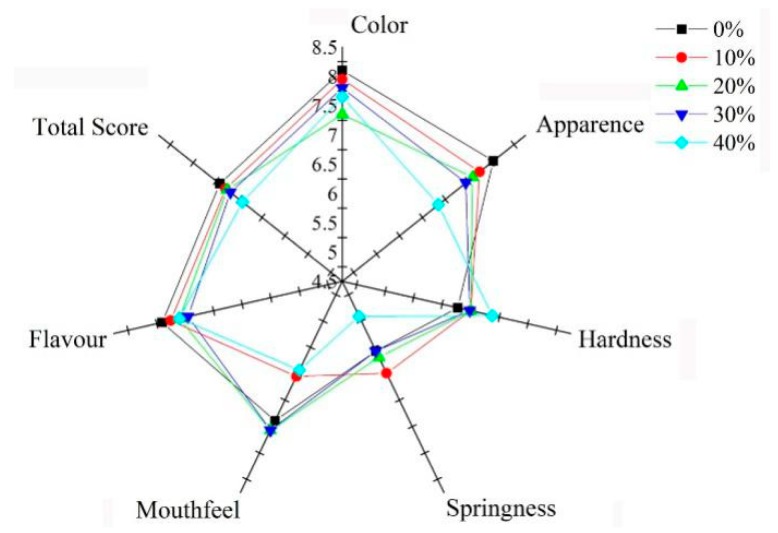
Changes in wheat flour noodles sensory evaluation as a function of Chinese yam powder. The digital number present the final result in ten-point system. Each coordinate axis has the same scale.

The CYP-fortified cooked noodles were evaluated for their sensory properties and the results are presented in Figure 3. The result of sensory evaluation showed that different addition of CYP affected the sensory attributes of wheat flour noodles. Compared with wheat flour noodles, CYP exhibited obvious low scores for appearance, color, flavor, and total score. The results showed that CYP supplementation had negatively affected appearance, color, and flavor due to yam characteristic flavor and color which is easier to be oxidized.

Up to 20% concentration of CYP, some sensory properties were satisfactory such as hardness, mouthfeel, and springiness, which was also revealed in TPA and SEM properties. Incorporation of more than 20% of CYP resulted in low total score and the noodles were not acceptable due to diluted gluten. Similarly, in an earlier study by Sanju and Kawaljit [20], up to 7% incorporation of fenugreek flour into wheat flour resulted into a satisfactory score for noodles, but more than 7% addition lowered the score and the noodles were not acceptable.

### 3.8. Microstructure

**Figure 4:**
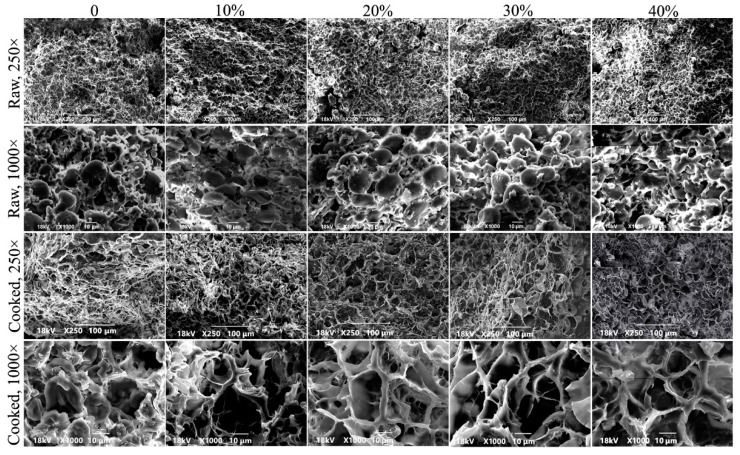
SEM micrographs of wheat flour noodles supplemented with different levels of Chinese yam powder.

The SEM microstructures of raw noodles and cooked noodles are shown in Figure 4 explaining the texture of noodles which can provide the supportive information for the result of cooking properties. Gluten and starch play the important role to support the protein network which is responsible for the stability [34]. It can be seen from the Figure 4 that, for raw noodles prepared from wheat flour supplemented with CYP, starch particles were more closely bound to gluten proteins, and the surface was relatively smooth and flat without holes. The addition of yam flour enhanced the binding ability of starch particles and gluten proteins in the dough. This may be due to the colloidal properties of yam flour, which allow starch particles to be more firmly filled in the gluten network [35]. Therefore, it can make starch and protein more closely bound together and result in a rigid noodle texture. From Figure 4, it can be revealed that after boiling and drying, many starch particles were exposed in the noodles with irregular shape (mostly oval), different sizes, and distributed in the gluten network in an uneven way. Flaky noodles with irregular holes and loose texture were noticed which was consistent with a previous study where excessive konjac could limit starch swelling, decrease spontaneous rupture, and accelerate the fragile network structure [31].

## 4. Conclusions

Our study explored the possibility of preparing noodles with acceptable quality from wheat flour supplemented with different levels of Chinese yam powder. Rheological, farinograph, and gelatinization texture properties were evaluated. It can be concluded that the addition of CYP to wheat flour can affect the overall properties of dough and noodles. When supplemented, the texture of noodles as evidenced by several parameters such as water absorption, cooking loss and firmness were improved. For sensory evaluation, some properties such as hardness, mouthfeel, and springiness were improved up to 20% incorporation of CYP, which supported the TPA properties. For microstructure, SEM revealed that addition of CYP could promote the close binding of starch particles to gluten protein and thereby resulted in relatively smooth surface. The above results encourage further studies to confirm the beneficial effects in terms of improved nutritional values of the noodles prepared from wheat flour supplemented with acceptable levels of CYP. They can in fact be advantageous for consumers seeking alternatives containing natural healthy ingredients in foods. The results of the current study demonstrate the potential for extending the use of Chinese yam powder in wheat noodle for industrial application.

## Figures and Tables

**Table 1:** Chemical composition of wheat flour–yam powder mixtures.

Chinese Yam Powder Concentration (%)	Moisture (g/100 g)	Ash (g/100 g)	Protein (g/100 g)	Fat (g/100 g)	Starch (g/100 g)	Gluten (g/100 g)
0	8.98 ± 0.051 ^a^	0.61 ± 0.057 ^a^	8.55 ± 0.015 ^a^	1.38 ± 0.087 ^a^	68.3 ± 0.32 ^a^	32.07 ± 0.068 ^a^
10	8.88 ± 0.071 ^b^	0.61 ± 0.0057 ^a^	8.97 ± 0.035 ^b^	1.25 ± 0.020 ^a^	68.7 ± 0.11 ^a^	28.72 ± 0.56 ^b^
20	8.27 ± 0.030 ^c^	0.61 ± 0.0003 ^a^	9.56 ± 0.026 ^c^	1.06 ± 0.015 ^b^	69.08 ± 0.065 ^ab^	25.34 ± 0.37 ^c^
30	7.12 ± 0.049 ^d^	0.61 ± 0.0033 ^a^	9.85 ± 0.045 ^c^	1.04 ± 0.026 ^b^	69.51 ± 0.12b ^c^	24.05 ± 0.15 ^c^
40	6.68 ± 0.031 ^e^	0.62 ± 0.0057 ^a^	10.56 ± 0.025 ^d^	0.91 ± 0.014 ^c^	70.12 ± 0.043 ^c^	18.06 ± 0.11 ^d^
100	5.96 ± 0.019 ^f^	0.62 ± 0.0064 ^a^	12.49 ± 0.015 ^e^	0.56 ± 0.0088 ^d^	72.20 ± 0.13 ^d^	0

Data were expressed as means ± standard deviations (*n* = 3). Values with different letters are significantly different (*p* < 0.05).

**Table 2:** Gelatinization and farinograph properties of wheat flour–yam powder mixtures.

CYP Concentration (%)	Gelatinization Properties				Farinograph Properties			
	Onset Gelatinization Temperature (°C)	Conclusion Gelatinization Temperature(°C)	Gelatinization Temperature Peak (°C)	Gelatinization Temperature Range (°C)	Water Absorption (%)	Dough Development Time (min)	Dough Stability Time (min)	Degree of Softening (BU)
0	59.42 ± 0.22 ^a^	72.24 ± 0.21 ^a^	61.10 ± 0.09 ^a^	12.82 ± 0.05 ^a^	56.2 ± 0.2 ^a^	2.2 ± 0.004 ^a^	4.8 ± 0.01 ^a^	90 ± 0.45 ^a^
10	60.77 ± 0.35 ^b^	71.12 ± 015 ^b^	63.67 ± 0.06 ^b^	10.35 ± 0.04 ^b^	57.0 ± 0.4 ^b^	2.2 ± 0.002 ^a^	3.7 ± 0.007 ^b^	178 ± 0.24 ^b^
20	59.58 ± 0.23 ^ab^	71.29 ± 0.19 ^a^	63.60 ± 0.13 ^b^	11.71 ± 0.04 ^c^	58.1 ± 0.1 ^c^	2.4 ± 0.005 ^b^	3.3 ± 0.005 ^c^	172 ± 0.27 ^b^
30	60.14 ± 0.14 ^b^	72.03 ± 0.22 ^a^	63.84 ± 0.08 ^b^	11.89 ± 0.05 ^d^	58.8 ± 0.2 ^c^	2.9 ± 0.004 ^c^	3.1 ± 0.002 ^d^	162 ± 0.19 ^c^
40	63.46 ± 0.19 ^c^	73.64 ± 0.23 ^a^	67.21 ± 0.04 ^c^	10.18 ± 0.03 ^e^	55.9 ± 0.1 ^d^	2.4 ± 0.01 ^b^	5.6 ± 0.02 ^e^	68 ± 0.34 ^d^
100	72.45 ± 0.18 ^d^	82.68 ± 0.16 ^c^	76.38 ± 0.02 ^d^	10.23 ± 0.06 ^e^				

Data were expressed as means ± standard deviations (*n* = 3). Values with different letters are significantly different (*p* < 0.05).

## Supplementary Materials

The following are available online at https://www.mdpi.com/2304-8158/8/10/519/s1, Figure S1: Differential scanning calorimetry (DSC) traces of the gelatinization parameters of control wheat flour (W) and yam powder (Y) and wheat flour-yam powder mixtures at 10% to 40%.
