# Supplementary material for: Microstructural, Textural, Sensory Properties and Quality of Wheat–Yam Composite Flour Noodles
Source: Foods. 2019 Oct 21;8(10):519. doi: 10.3390/foods8100519 (PMC6835949; doi:10.3390/foods8100519)
Supplement: Supplementary file 1 [file foods-08-00519-s001.pdf]

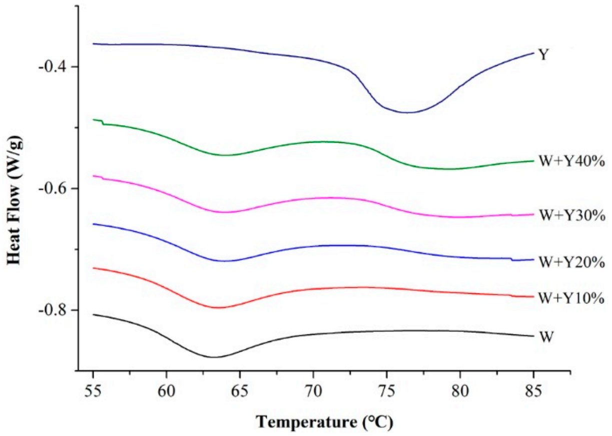

Figure S1. Differential scanning calorimetry (DSC) traces of the gelatinization parameters of control wheat flour (W) and yam powder (Y) and wheat flour-yam powder mixtures at 10% to 40%.
